# The IMD and Toll canonical immune pathways of *Triatoma pallidipennis* are preferentially activated by Gram-negative and Gram-positive bacteria, respectively, but cross-activation also occurs

**DOI:** 10.1186/s13071-022-05363-y

**Published:** 2022-07-12

**Authors:** Alvarado-Delgado Alejandro, Juárez-Palma Lilia, Maritinez-Bartneche Jesús, Rodriguez Mario Henry

**Affiliations:** Centro de Investigación Sobre Enfermedades Infecciosas, Instituto Nacional de Salud Pública, Av. Universidad 655, CP 62100 Cuernavaca, Morelos México

**Keywords:** *T. pallidipennis*, Tp*pgrp-lc*, Tp*toll*, Tp*relish*, IMD pathway

## Abstract

**Background:**

Antimicrobial peptides (AMPs) participate in the humoral immune response of insects eliminating invasive microorganisms. The immune deficiency pathway (IMD) and Toll are the main pathways by which the synthesis of these molecules is regulated in response to Gram-negative (IMD pathway) or Gram-positive (Toll pathway) bacteria. Various pattern-recognition receptors (PRRs) participate in the recognition of microorganisms, such as *pgrp-lc* and *toll*, which trigger signaling cascades and activate NF-κB family transcription factors, such as *relish*, that translocate to the cell nucleus, mainly in the fat body, inducing AMP gene transcription.

**Methods:**

*T. pallidipennis* inhibited in Tp*pgrp-lc*, Tp*toll*, and Tp*relish* were challenged with *E. coli* and *M. luteus* to analyze the expression of AMPs transcripts in the fat body and to execute survival assays.

**Results:**

In this work we investigated the participation of the *pgrp-lc* and *toll* receptor genes and the *relish* transcription factor (designated as Tp*pgrp-lc*, Tp*toll*, and Tp*relish*), in the transcriptional regulation of *defensin B*, *prolixicin*, and *lysozyme B* in *Triatoma pallidipennis*, one of the main vectors of Chagas disease. AMP transcript abundance was higher in the fat body of blood-fed than non-fed bugs. Challenge with *Escherichia coli* or *Micrococcus luteus* induced differential increases in AMP transcripts. Additionally, silencing of Tp*pgrp-lc*, Tp*toll*, and Tp*relish* resulted in reduced AMP transcription and survival of bugs after a bacterial challenge.

**Conclusions:**

Our findings demonstrated that the IMD and Toll pathways in *T. pallidipennis* preferentially respond to Gram-negative and Gram-positive bacteria, respectively, by increasing the expression of AMP transcripts, but cross-induction also occurs.

**Graphical Abstract:**

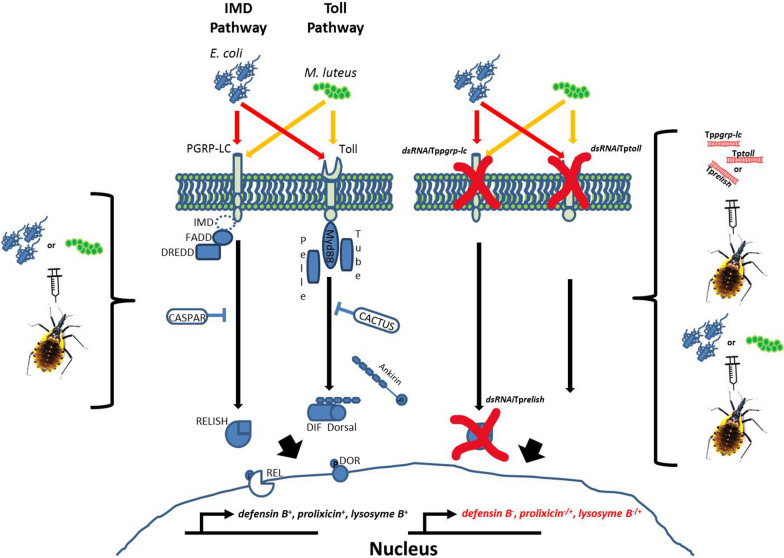

**Supplementary Information:**

The online version contains supplementary material available at 10.1186/s13071-022-05363-y.

## Background

Triatominae (Hemiptera: Reduviidae) are insects that transmit *Trypanosoma cruzi* parasites, which cause American trypanosomiasis [1]. In Mexico, *Triatoma pallidipennis* is an important vector [2–4]. Triatomines respond to microbial infections via cellular and humoral immune mechanisms. The humoral component comprises antimicrobial peptides (AMPs), lectins, and melanin through the pro-phenol oxidase (PPO) cascade [5–8]. AMPs are mainly produced in the fat body, the midgut, and hemocytes [9], following the activation of the immune deficiency (IMD) [10], Toll [11], and JAk-Stat pathways [12, 13]. The IMD is similar to the tumor necrosis factor receptor (TNFR) pathway in mammals [14]. The Toll pathway involves molecules with some parallels to mammalian signaling cascades like the interleukin-1 receptor (IL-1R) and the Toll-like receptors (TLRs) [15].

In *Drosophila melanogaster*, the IMD pathway [10] is activated when the diaminopimelic acid peptidoglycan of Gram-negative bacteria binds to the transmembrane receptor protein peptidoglycan recognition protein-long chain (PGRP-LC). This receptor recruits and activates the IMD, Fas-associated death domain protein (FADD), death-related ced-3/Nedd2-like caspase (DREDD), transforming growth factor-β-activated kinase-1 (TAK1), and IκB kinase (IKK) complex, which leads to the activation of the NF-kB family transcription factor Relish via the cleavage of DREDD [16]. The activated Relish moves into the nucleus and induces the expression of AMP genes such as cecropins, attacins, diptericins, drosomycin [17], and metchnikowin [18]. This pathway is negatively regulated by Caspar, which inhibits DREDD and prevents the translocation of Relish into the nucleus [19, 20].

The Toll pathway is activated when Lys-type peptidoglycan and β-1,3-glycan of Gram-negative bacteria induce the proteolytic cleavage of proSpätzle. Spätzle binds to the Toll receptor on the cell membrane [21], and this complex triggers a cytoplasmic signal transduction cascade through myeloid differentiation primary response protein (MyD88)-Tube-Pelle. Pelle phosphorylates and degrades Cactus, which releases Dorsal and Dif [22]. The translocation of the NF-kB family Dorsal and Dif into the nucleus induces the expression of the AMPs drosomycin, defensin 2, and metchnikowin [23].

Most of the knowledge regarding these immune pathways in insects has been obtained from studies of dipteran insects such as *D. melanogaster* and *Aedes aegypti* [24–26]. Observations indicate that these pathways can be synergetic [27–31]. For instance, the production of several AMPs, regardless of the bacterial challenge, in the hemipteran *Plautia stali* suggests an interaction between the IMD and Toll pathways [27].

Ortholog molecules associated with the IMD and Toll pathways, their corresponding pattern-recognition receptors (PRRs) such as *pgrp-lc* and *pgrp-la*, and AMPs have been identified in various triatomine species [32–36]. However, several components of the Imd cascade, including the IMD protein, appear to be absent or incomplete in these insects [33, 35], and this absence appears to be a common feature in insects with incomplete metamorphosis [35]. Despite the absence of key mediators, it has been documented in *Rhodnius prolixus* that the IMD pathway principally regulates AMP expression against Gram-negative, but also against Gram-positive bacteria [36], which suggests that in triatomines, the generation of AMPs may not follow the canonical IMD and Toll pathway activation.

In this work, we investigated the induction of Tp*pgrp-lc*, Tp*toll*, Tp*relish*, and immune response gene (IRG) transcription under the control of the IMD and Toll pathways in *T. pallidipennis* challenged with Gram-positive and Gram-negative bacteria. We observed that, although Gram-negative and Gram-positive bacteria preferentially activated the IMD and Toll pathways, respectively, cross-activation occurred, albeit with less intensity. This suggests that in *T. pallidipennis*, as in other hemimetabolic insects [27], cross-activation of immune pathways could occur in response to systemic infections.

## Methods

### Insect rearing

*Triatoma pallidipennis* nymphs were obtained from a colony established with specimens collected from Chalcatzingo, Jantetelco Morelos, Mexico, in the insectary of the National Institute of Public Health. Insects were maintained at 28°C and 70–80% relative humidity under a photoperiod of 12 h light and 12 h dark. They were fed rabbit blood 10 days after molting, using artificial feeders. All experiments were conducted using 10 days-post-feeding fifth-instar nymphs. The protocols were approved by the Biosafety, Ethics and Research Committees of the National Institute of Public Health, CB17-229, CB: 1491, CI: 1500.

### Blood-fed and non-fed insects

Groups of 10 newly emerged fifth-instar nymphs were fed rabbit blood ad libitum and were maintained for 10 days under insectary conditions; only fully engorged bugs were used in experiments. In addition, groups of 10 non-fed nymphs were maintained under the same conditions and used as control in initial experiments. After identifying that the expression of AMPs was higher in blood-fed insects, RNA interference experiments and survival assays were performed only with blood-fed bugs.

### Bacterial challenge and fat body isolation

Gram-positive *Micrococcus luteus* (Sigma-Aldrich, M-0508) and Gram-negative *Escherichia coli* bacteria (8739 strain atcc.org/products) were cultured overnight at 30 °C and 37 °C, respectively, in Luria–Bertani broth in tubes rotating at 200 rpm. On the next day, 100 µl of each culture was inoculated in 5 ml of Luria–Bertani broth and incubated under the above-mentioned conditions for approximately 3 h until they reached a density of 0.7 (OD^600^). The liquid cultures were centrifuged, and the pellets were washed with 250 µl of phosphate-buffered saline (PBS) (137 mM NaCl, 2.7 mM KCl, 10 mM sodium phosphate, pH7.2).

Groups of eight nymphs were cold-anesthetized (4 °C), and 2 × 10^6^ colony-forming units (CFU) (20 μl PBS) of live *M. luteus* or *E. coli* were injected through the interstitial integument between the abdomen and thorax cuticle using a Hamilton syringe. Control groups were injected with 20 μl of sterile PBS or non-challenged. Twenty-four hours later, insects were dissected and their fat body tissues were recuperated in PBS. Tissue samples were stored in 200 μl TRIzol (Thermo Fisher Scientific, Waltham, MA, USA) at −70 °C until processing for quantitative real-time polymerase chain reaction (qPCR) estimation of AMP transcripts. Each treatment had three replicates per group.

### RNA extraction and complementary DNA (cDNA) synthesis

Total RNA from fat body tissues was extracted using TRIzol (Thermo Fisher Scientific, Waltham, MA, USA) following the manufacturer’s recommendations. Briefly, about 50 mg of fat body samples collected in 200 μl TRIzol in Eppendorf tubes (Thermo Fisher Scientific) was macerated in a biovortex with four pulses/min with 30 s between pulses. After adding 20 μl of chloroform (Sigma-Aldrich, St. Louis, MO, USA), the preparations were mixed and centrifuged for 15 min at 10,000×*g* at 4 °C. The aqueous phase was recovered and 250 μl of cold isopropanol (Sigma-Aldrich) was added, mixed, and incubated at −20 °C for 1 h. The samples were centrifuged at 10,000×*g* for 10 min, and the pellets were washed with 500 μl 75% ethanol and centrifuged at 7000×*g* for 5 min. The supernatants were removed, and the pellets were suspended in 40 μl diethyl pyrocarbonate (DEPC, Sigma-Aldrich)-treated water. RNA was quantified with a NanoDrop 1000 spectrophotometer v. 3.7 (Thermo Fisher Scientific) and visualized using electrophoresis in agarose gels stained with EpiQuik DNA stain (EpiGentek, Farmingdale, NY, USA).

Five micrograms of total RNA was treated with four units of DNAse I (Thermo Fisher Scientific, Waltham, MA, USA) for 30 min at 37 °C, and subsequently inactivated at 75 °C for 15 min. First-strand cDNA synthesis was performed in 25 μl reactions containing 2 μg total RNA using an oligo dT primer (Thermo Fisher Scientific) with SuperScript^®^ IV Reverse Transcriptase (RT) (Thermo Fisher Scientific) synthesis reactions. The preparations were incubated for 1 h at 42 °C, and the RT enzyme was inactivated at 75 °C for 15 min. The synthesized cDNA was diluted 1:20 with DEPC water and stored at −70 °C until use.

### PCR of Tp*pgrp*, Tp*toll*, Tp*relish*, and AMP transcripts

The transcription of Tp*pgrp-lc,* Tp*toll*, Tp*relish*, *defensin B*, *lysozyme B*, and *prolixicin* was investigated in cDNA templates by RT-PCR, using the *T. pallidipennis* *β-actin* gene as control. Oligonucleotides were designed using previously identified transcriptome sequences of *T. pallidipennis* (Tp*pgrp-lc*: TPAL_isotig03340; Tp*toll*: TPAL_H9TUR5Q01DQBBI; Tp*relish*: TPAL_H9TUR5Q02INIGT; *prolixicin*: TPAL_isotig05995, *defensin B*: TPAL_H9TUR5Q02J2RC5; *lysozyme B*: TPAL_isotig04641; *β-actin*: TPAL_H9TUR5Q01CBM3V) [35] (Additional file 1: Table S1).

The identity of each sequence was confirmed by analyzing each transcript with its orthologous genes in *R. prolixus*, *Triatoma brasiliensis*, *D. melanogaster*, *Reticulitermes speratus*, *Coptotermes formosanus*, *P. stali*, and *Cimex lectularius*. Domains associated with the main functions were identified using InterPro version 87.0 [37]. All PCR reactions used 1 U DreamTaq Polymerase (Thermo Fisher Scientific, Waltham, MA, USA), 0.5 mM dNTP mix, 1 mM MgCl_2_, 0.5 pmol of each oligonucleotide, and 3 µl of cDNA. The cycling conditions were as follows: denaturation at 95 °C for 3 min and 35 cycles of denaturation at 95 °C for 30 s, annealing at 54 °C for 30 s (Tp*toll*, Tp*relish*, and *β-actin*), 58 °C for 30 s (Tp*pgrp-lc*, *lysozyme B*, *prolixicin*, and *defensin B*), and extension at 72 °C for 1 min. The obtained amplicons were sequenced and their identity analyzed.

### Quantitative real-time PCR

We used qPCR to analyze the expression of Tp*pgrp-lc* and Tp*toll* receptors and Tp*relish* as well as of *prolixicin*, *defensin B*, and *lysozyme B* in individual cDNA samples of fat body tissue after the challenge with *M. luteus* and *E. coli*. Each reaction was performed in a final volume of 10 µl, containing 1 µl of cDNA (1:20), 1.5 pmol of each oligonucleotide, and 5 µl of SYBR Green 2X Mix (NZY qPCR Green Master Mix, nzytech, Lisbon, Portugal). qPCR was performed on a Rotor-Gene Q 5plex (Qiagen, Hilden, Germany). The amplification efficiency for each transcript was analyzed (by serial dilutions of the cDNA sample) using the standard curve method, with the formula *E* = 10(−1/slope) − 1 (*r* = 0.94). The qPCR conditions used were as follows: 95 °C for 3 min, 40 cycles of 95 °C for 15 s and 61 °C for 1 min, followed by melt curve analysis to confirm the specificity of the reaction and 1.2% agarose gel electrophoresis to determine the molecular weight. Controls without templates were included with each primer set, to verify the absence of exogenous DNA and oligonucleotide dimers.

### Double-stranded (ds)RNA target selection and synthesis

To analyze the participation of Tp*pgrp-lc*, Tp*toll*, and Tp*relish* in the AMP transcript synthesis, we knocked down their transcript translation using the transcriptAid T7 High Yield Transcription Kit (Thermo Fisher Scientific cat. #K0441, Waltham, MA, USA). The oligonucleotides used to generate dsRNA of Tp*pgrp-lc*, Tp*toll*, and Tp*relish* were flanked by the T7 promoter GTAATACGACTCACTATAGGG sequence at the 5′end (Additional file 1: Table S1). To reduce the off-target silencing, the region with the lowest number of potential off-target silencing fragments was selected to amplify two fragments of 635 base pairs (bp) (Tp*pgrp-lc*), 391 bp (Tp*toll*), and 170 bp (Tp*relish*), respectively. Each fragment was initially amplified by PCR with oligonucleotides that did not include the promoter sequence to the T7 RNA polymerase (RNApol). These products served as template DNA to amplify the above-mentioned products and to integrate the T7 promoter sequence recognized by T7 RNA polymerase. These were used as templates to synthesize dsRNA, according to the vendor’s recommendations (Thermo Fisher Scientific). The dsRNA was precipitated with ethanol, visualized in 1% agarose gel, and quantified using a NanoDrop 1000 spectrophotometer v. 3.7 (Thermo Fisher Scientific). A sequence coding for a 2223-nucleotide runoff transcript included in the TranscriptAid T7 High Yield Transcription Kit (Thermo Fisher Scientific cat. #K0441) was used as a negative control in RNA interference experiments.

### RNA interference experiments

The inhibition kinetics of gene silencing were evaluated for each transcript using groups of 12 fifth-instar nymphs. Two micrograms of dsRNA Tp*pgrp-lc*, Tp*toll*, and Tp*relish* were each suspended in 20 µl NaCl 0.137 M, KCl 0.0027 M, Na_2_HPO_4_ 0.01 M, KH_2_PO_4_ 0.0018 M, pH7.4, and injected into the insects of each group using a Hamilton syringe. Inoculated insects were kept under insectary conditions. The fat body was removed from three specimens of each group at 5, 7, 11, and 15 days post-inoculation, and the expression of each gene was analyzed by qPCR (Rotor-Gene Q, Qiagen) (Additional file 3: Figure S1). As endogenous control, we used the *β-actin* gene, which was the most stable in *T. pallidipennis* fat body samples. Next, 12 groups of 10 fifth-instar nymphs were inoculated with dsRNA Tp*pgrp-lc*, Tp*toll*, Tp*relish*, or *irrelevant dsRNA* as control (Thermo Fisher Scientific cat. #K0441, Waltham, MA, USA), as described above. On day 15 (Tp*pgrp-lc* and Tp*relish*) or 7 (Tp*toll*) post-inoculation, they were challenged with 2 × 10^6^ CFU (20 μl PBS) of live *M. luteus*, *E. coli*, or sterile PBS, as described above. Twenty-four hours post-challenge, their fat body tissues were recuperated and used to estimate the transcription of Tp*pgrp-lc*, Tp*toll*, Tp*relish*, *prolixicin*, *defensin B*, *lysozyme B*, and *β-actin*. The experimental groups, treatment, and bacterial challenge are presented in Additional file 2: Table S2. Each treatment had three biological replicates per group.

### Survival assays in Tp***relish***-, Tp***pgrp-lc***-, and Tp***toll***-inhibited insects

Ten days after blood-feeding, 15 groups of 40 fifth-instar nymphs were inoculated with 2 µg double-stranded RNA. Three groups each received *irrelevant dsRNA*, *ds relish*, *ds*Tp*pgrp-lc*, *ds*Tp*toll*, or both (Tp*pgrp-lc*-Tp*toll*). One member of each group remained unchallenged, while another was injected with *E. coli* and the other with *M. luteus*. Bacterial challenges were performed by intrathecal injection at 15 or 7 days post-dsRNA inoculation. The survival of the bugs was recorded every day up to 30 days after bacterial challenge (experimental groups, treatment, and time of bacterial challenge after dsRNA inoculation are presented in Additional file 2: Table S2).

### Statistical analysis

The relative differences in the expression of transcripts were calculated using the 2^–ΔΔCt^ method [38]. As endogenous control, we used the *β-actin* gene. The values obtained from the ∆Ct analysis (Ct_value of problem transcript_ − Ct_value of *β-actin*_) were used to compare each transcript between groups (∆∆Ct) in all experiments (e.g., ∆C_prolixicin_ group blood-fed − ∆C_prolixicin_ group non-fed), and Kruskal–Wallis tests were performed to determine differences in gene expression between each treatment and their controls. Graphs were made using GraphPad Prism 6. *P*-values of *P* < 0.05 were considered significant. Groups to compare the fold expression were as follows: analysis of transcript expression in blood-fed bugs (blood-fed group vs. non-fed group), analysis of transcript expression in blood-fed and challenged bugs (blood-fed and challenged group vs. PBS group normalized with blood-fed and unchallenged group), and analysis of transcript expression in interfered and challenged bugs (blood-fed, interfered, and challenged group vs. blood-fed, *irrelevant dsRNA* inoculated challenged and normalized with unchallenged group). Percent survival and a Kaplan–Meier survival plot were realized using GraphPad Prism 6 and Kaplan–Meier log-rank and Wilcoxon–Gehan analysis.

## Results

The expression of Tp*pgrp-lc*, Tp*toll*, Tp*relish*, *defensin B*, *prolixicin*, and *lysozyme B* transcripts in the fat body of *T. pallidipennis* was confirmed by PCR, followed by sequencing and amino acid alignment of each amplified fragment (Additional file 4: Figure S2).

### Expression of Tp*pgrp-lc*, Tp*toll*, Tp*relish*, and AMP transcripts was higher in blood-fed than in non-fed non-challenged insects

**Fig. 1:**
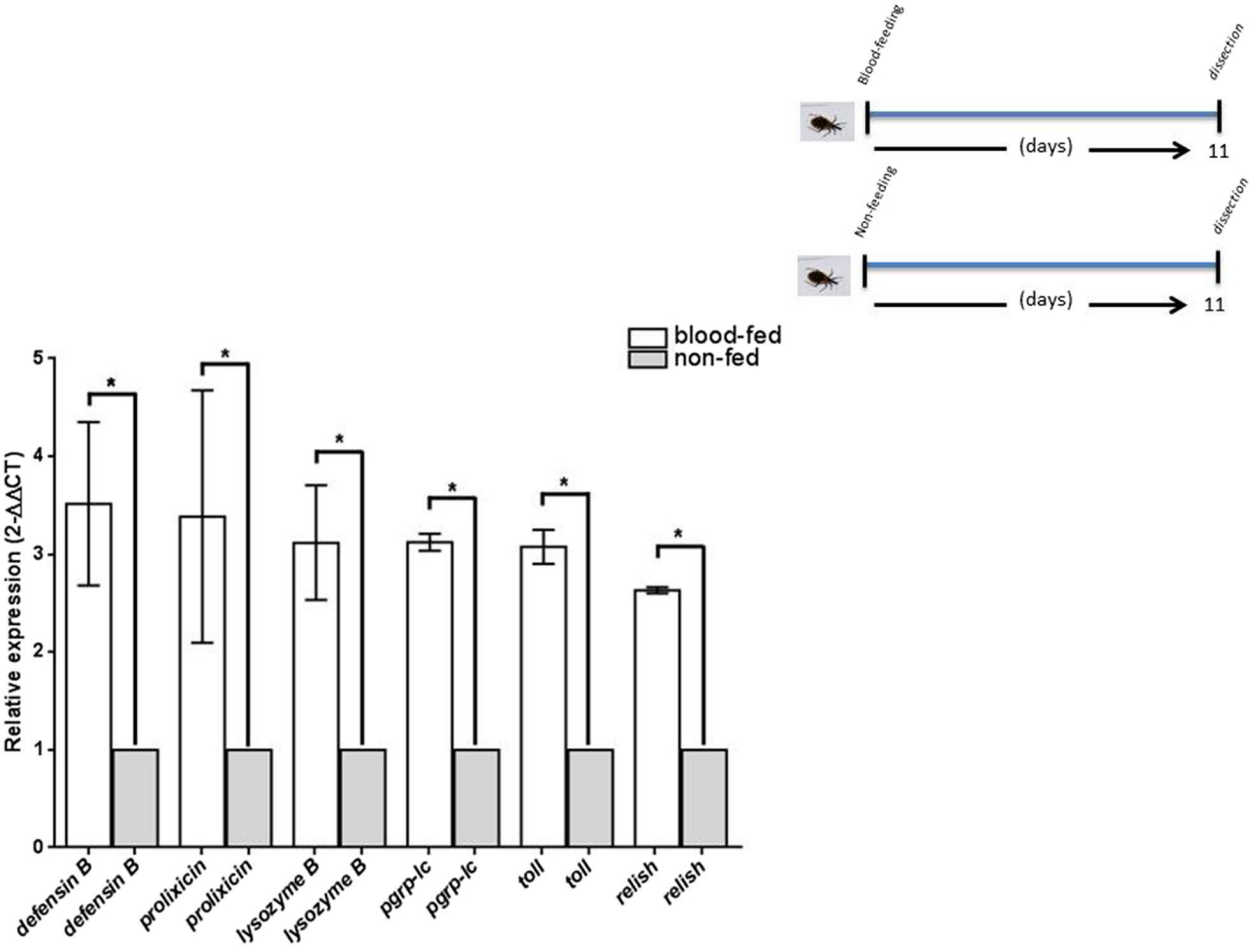
Relative expression of Tp*pgrp*-*lc*, Tp*toll*, Tp*relish*, and antimicrobial peptide transcripts in blood-fed, non-fed, and non-challenged bugs. All transcripts in the fat body of blood-fed bugs showed significantly higher expression (*defensin B* 3.51-, *prolixicin* 3.38-, *lysozyme B* 3.12-, Tp*pgrp*-*lc* 3.12-, Tp*toll* 3.08-, and Tp*relish* 2.63-fold), compared with non-fed groups. Groups with three biological replicas of eight bugs were analyzed. Relative expression (2^−∆∆CT^) is the quantified change between transcripts. Asterisks indicate *P* < 0.0001. Bars represent the mean transcript levels ± 95% CI. All groups were adjusted for *β-actin*. Upper right: timeline showing the experimental strategy to obtain the fat body of each bug in the non-fed and blood-fed groups

In all non-challenged insects, the expression of all transcripts was higher in blood-fed than in non-fed fat body samples, including Tp*pgrp-lc* (3.12-fold, SE 3.03–3.20 *P* < 0.0001), Tp*toll* (3.08-fold, SE 2.97–3.28 *P* < 0.0001), Tp*relish* (2.63-fold, 2.61–2.67 *P* < 0.0001), *defensin B* (3.51-fold, SE 2.67–4.34 *P* < 0.0001), *prolixicin* (3.38-fold, SE 2.34–4.83 *P* < 0.0001), and *lysozyme B* (3.12-fold, SE 2.45–3.54 *P* < 0.0001) (Fig. 1, Table 1).

**Table 1:** Relative expression of transcripts analyzed in this work

Transcript	*Pgrp-lc*	*Toll*	*Relish*	*Defensin B*	*Prolixicin*	*Lysozyme B*
Non-fed/unchallenged	1	1	1	1	1	1
Fed/unchallenged	**3.12 (3.03–3.20) *P* < 0.0001**	**3.08 (2.97–3.28) *P* < 0.0001**	**2.63 (2.61–2.67) *P* < 0.0001**	**3.51 (2.67–4.34) *P* < 0.0001**	**3.38 (2.34–4.83) *P* < 0.0001**	**3.12 (2.45–3.54) *P* < 0.0001**
Non-fed-PBS	1	1	1	1	1	1
Non-fed/*E. coli*	**1.35 (1.08–1.65) *P* < 0.05**	1.26 (0.83–1.98)	**1.53 (1.24–2.04) *P* < 0.05**	**2.74 (1.93–3.9) *P* < 0.05**	**2 (1.65—2.39) *P* < 0.05**	**1.72 (1.19–2.03) *P* < 0.05**
Non-fed/*M. luteus*	*0.86 (0.54–1.10)* *P* < *0.357*	1.53 (0.79–2.02)^a^	1.54 (0.84–1.91)^a^	1.78 (1.14–2.78)^a^	**2 (1.73—2.58) *P* < 0.05**	**1.23 (1.01–1.39) *P* < 0.05**
Fed-PBS	1	1	1	1	1	1
Fed/*E. coli*	**6.67 (5.99–7.37) *P* < 0.005**	**2.64 (1.98–3.04) *P* < 0.05**	**4.11 (3.67–4.37) *P* < 0.05**	**6.58 (3.36–8.65) *P* < 0.005**	**2.49 (1.93–2.96) *P* < 0.05**	**2.67 (2.43–3.04) *P* < 0. 05**
Fed/*M. luteus*	**3.62 (2.84–4.20) *P* < 0.05**	**15 (12.10–16-60) *P* < 0.0001**	**2.08 (1.87–2.48) *P* < 0.05**	**4.67 (3.63–6.14) *P* < 0.05**	**3.25 (2.41–3.94) *P* < 0.05**	**1.45 (1.24–1.67) *P* < 0.05**
Fed/*irrelevant ds*RNA				1	1	1
Fed/*pgrp*^−^/*E. coli*				**0.82 (0.63–1.06) *P* < 0.0357**	**0.6 (0.43–0.89) *P* < 0.0036**	**0.68 (0.62–0.77) *P* < 0. 0036**
Fed/*relish*^−^/*E. coli*				**24.59 (18–34.77) *P* < 0.0036**	**2.7 (1.34–3.94) *P* < 0. 0250**	**0.6 (0.16–1.08) *P* < 0.0375**
Fed/*toll*^−^/*E. coli*				1.46 (0.87–1.89) *P* < 0.7	**0.68 (0.51–0.79) *P* < 0.0036**	**0.95 (0.82–1.06) *P* < 0.0357**
Fed/*irrelevant ds*RNA				1	1	1
Fed/*pgrp*^−^/*M. luteus*				**0.34 (0.25–0.41) *P* < 0.0036**	**0.81 (0.76–0.84) *P* < 0.0036**	**0.26 (0.11–0.45) *P* < 0.0036**
Fed/*relish*^−^/*M. luteus*				**0.86 (0.72–1.03) *P* < 0.0357**	**1.49 (1.27–1.63) *P* < 0.0036**	**1.61 (1.33–2.11) *P* < 0.05**
Fed/*toll*^−^/*M. luteus*				**0.51 (0.38–1.31) *P* < 0.0036**	**2.32 (1.87–2.63) *P* < 0.0036**	**0.86 (0.67–1.02) *P* < 0.0357**

Transcripts that had a significant increase are shown in bold. Transcripts with no significant increase are underlined or no increase are in italics. Standard errors are shown in parentheses

^a^The comparison between these groups did not yield significant differences. P < 0.420

### Inoculation with *E. coli* and *M. luteus* increased the transcription of immune response genes in non-fed insects, and significant differences were observed in some transcripts after bacterial challenges

**Fig. 2:**
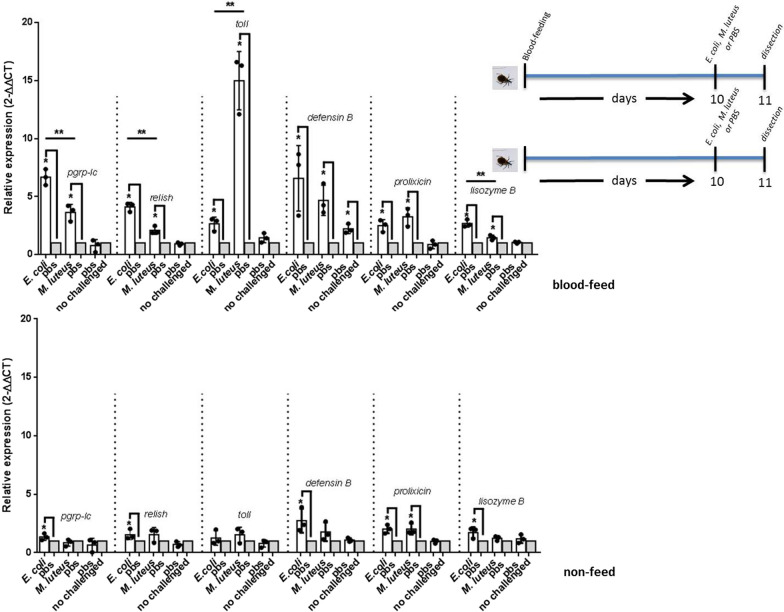
Relative expression of Tp*pgrp-lc*, Tp*toll*, and Tp*relish* and antimicrobial peptide transcripts in fat body of blood-fed and non-fed *T. pallidipennis* after immune challenge with *E. coli* and *M. luteus*. In blood-fed bugs, all transcripts increased significantly after challenge with *E. coli* or *M. luteus*. Tp*pgrp*-*lc* and Tp*relish* increased more with *E. coli* than with *M. luteus*, while Tp*toll* increased more with *M. luteus* than with *E.* challenge. *Defensin B* and *prolixicin* expression increased after challenge with *E. coli* or *M. luteus*, but these increases were not significant among groups. *Lysozyme* showed significantly higher expression against *E. coli* than against *M. luteus*. In the non-fed groups, the increases were lower than in blood-fed groups. Relative expression (2^−∆∆CT^) is the quantified change between transcripts, asterisks indicate *P* < 0.05, bars represent the mean transcript levels ± 95% CI, points represent the analyzed groups, and a double asterisk with a bar represents the significance (*P* < 0.05) of expression between the challenged groups, PBS group as control, and this normalized with the blood-fed and unchallenged group adjusted for *β-actin*. Upper right: timeline showing the experimental strategy to obtain the fat body of each bug in the non-fed and blood-fed groups

* Prolixicin* and *lysozyme B* transcript expression increased significantly in non-fed insects challenged with *E. coli* (2-fold, SE 1.65–2.39 *P* < 0.05 and 1.72-fold, SE 1.19–2.03 *P* < 0.05, respectively) and *M. luteus* (2-fold, SE 1.73–2.58 *P* < 0.05 and 1.23-fold, SE 1.01–1.39 *P* < 0.05, respectively), while *defensin B* (2.74-fold, SE 1.93–3.9 *P* < 0.05), Tp*pgrp-lc* (1.35-fold, SE 1.08–1.65 *P* < 0.05), and Tp*relish* (1.53-fold, SE 1.24–2.04 *P* < 0.05) increased after *E. coli* injections. However, no significant differences were observed in transcript expression between the two groups challenged with the two bacteria (Fig. 2, Table 1).

### Inoculation with *E. coli* and *M. luteus* induced differential transcript expression in blood-fed insects

Tp*pgrp-lc*, Tp*toll*, Tp*relish*, *defensin B*, *prolixicin*, and *lysozyme B* transcripts increased significantly in blood-fed insects challenged with *E. coli* and *M. luteus* (Fig. 2, Table 1). But Tp*pgrp-lc* and Tp*relish* transcripts increased more in insects challenged with *E. coli* (6.67-fold, SE 5.99–7.37 *P* < 0.005 and 4.11-fold, SE 3.67–4.37 *P* < 0.05, respectively) than in those challenged with *M. luteus* (3.62-fold, SE 2.84–4.20 *P* < 0.05 and 2.08-fold, SE 1.87–2.48 *P* < 0.05, respectively). While Tp*toll* transcript increased more in insects challenged with *M. luteus* (15-fold, SE 12.10–16.60 *P* < 0.0001) than in those challenged with *E. coli* (2.64-fold, SE 1.98–3.04 *P* < 0.05). *Lysozyme B* transcript increased more in insects challenged with *E. coli* (2.67-fold, SE 2.43–3.04 *P* < 0.05) than in those challenged with *M. luteus* (1.45-fold, SE 1.24–1.67 *P* < 0.05). While *defensin B* increased in insects challenged with *E. coli* (6.58-fold, SE 3.36–8.65 *P* < 0.005) and *M. luteus* (4.67-fold, SE 3.63–6.14 *P* < 0.05) and *prolixicin* transcription increased in insects challenged with *E. coli* (2.49-fold, SE 1.93–2.96 *P* < 0.05) and *M. luteus* (3.25-fold, SE 2.41–3.94 *P* < 0.05) (Fig. 2).

### Silencing of Tp*pgrp-lc* in *E. coli*- or *M. luteus*-challenged bugs inhibited the expression of antimicrobial transcripts, but silencing of Tp*toll* and Tp*relish* had a specific effect on *defensin B*, *prolixicin*, and *lysozyme B* transcripts

**Fig. 3:**
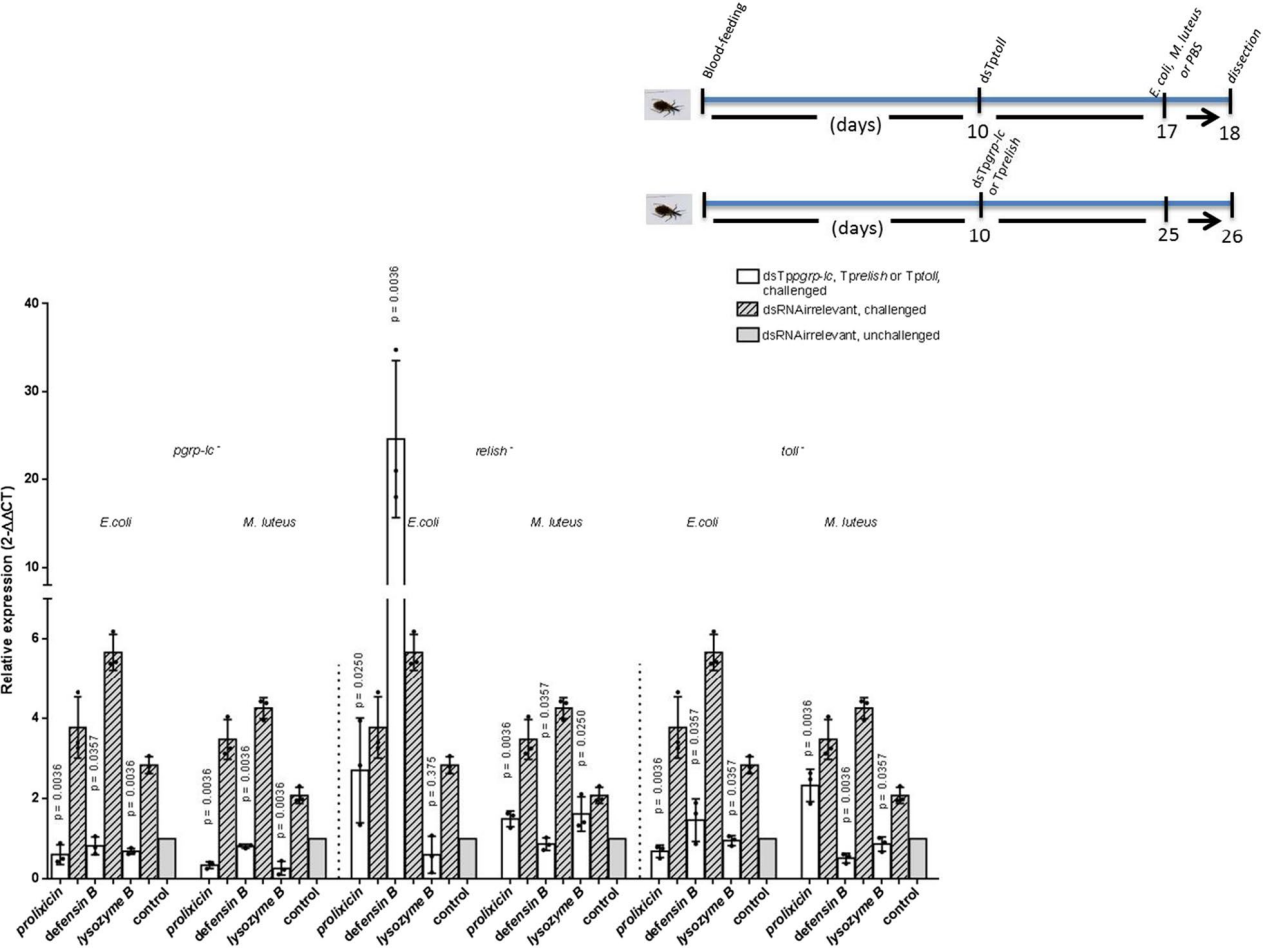
Relative expression of *prolixicin*, *defensin B*, and *lysozyme B* transcripts in the fat body after a challenge with *E. coli* or *M. luteus* of Tp*pgrp-lc*-, Tp*relish*-, and Tp*toll*-inhibited *T. pallidipennis*. After inhibiting Tp*pgrp-lc* or Tp*toll*, the expression of the three AMP transcripts decreased despite the challenge with *E. coli* or *M. luteus*. However, in bugs inhibited in Tp*relish*, *lysozyme B* and *prolixicin* transcripts decreased and *defensin B* transcripts continued to be expressed after challenge. These results confirm the specific participation of Tp*pgrp-lc* and Tp*toll* in the synthesis of some AMPs after a bacterial challenge. Relative expression (2^−∆∆CT^) is the quantified change between transcripts. Significant differences *P* < 0.05 are indicated, bars represent the mean transcript levels ± 95% CI, and points represent the analyzed groups. Upper right: timeline showing the experimental strategy to obtain the fat body of each bug in blood-fed, inhibited and challenged groups

To investigate the participation of the Tp*pgrp-lc* and Tp*toll* receptors and the Tp*relish* transcription factor in blood-fed insects, these genes were silenced up to 90% after 7 (Tp*toll*) or 15 days (Tp*pgrp*-*lc* and Tp*relish*) post-dsRNA inoculation (Additional file 3: Figure S1). Silencing of Tp*pgrp-lc* in insects challenged with *E. coli* inhibited the expression of *prolixicin*, *defensin B*, and *lysozyme B* transcripts (0.60-fold, SE 0.43–0.89 *P* < 0.0036; 0.82-fold, SE 0.63–1.06, *P* < 0.0357 and 0.68-fold, SE 0.62–0.77, *P* < 0.0036, respectively) (Fig. 3). In Tp*relish*-silenced insects, the expression of *lysozyme B* was inhibited (0.60-fold, SE 0.16–1.08, *P* < 0.0.375), but *defensin B* and *prolixicin* transcripts increased (24.59-fold, SE 18–34.77, *P* < 0.0036 and 2.70-fold, SE 1.34–3.94, *P* < 0.0250, respectively) (Fig. 3). In Tp*toll*-inhibited insects, the expression of *prolixicin* (0.68-fold, SE 0.51–0.79, *P* < 0.0036) and *lysozyme B* (0.95-fold, SE 0.82–1.06, *P* < 0.0357) decreased, and *defensin B* increased (1.46-fold, SE 0.87–1.89 *P* < 0.70) (Fig. 3).

Silencing of Tp*pgrp-lc* in *M. luteus*-challenged insects inhibited the expression of the *defensin B*, *prolixicin*, and *lysozyme B* transcripts (0.34-fold, SE 0.25–0.41, *P* < 0. 0036; 0.81-fold, SE 0.76–0.84 *P* < 0.0036 and 0.26-fold SE 0.11–0.45, *P* < 0.0036, respectively) (Fig. 3). In Tp*relish*-silenced bugs, *defensin B* transcript expression was inhibited (0.86-fold SE 0.72–1.03 *P* < 0.0357), while *prolixicin* and *lysozyme B* transcripts increased (1.49-fold SE 1.27–1.63, *P* < 0.0036 and 1.61-fold, SE 1.33–2.11, *P* < 0.05, respectively) (Fig. 3). In Tp*toll*-silenced bugs, *defensin B* and *lysozyme B* transcript was inhibited (0.51-fold, SE 0.38–1.31, *P* < 0.0036 and 0.86-fold, SE 0.67–1.02, *P* < 0.00357, respectively), and their expression was similar to that of bugs treated with an *irrelevant* *dsRNA*, while *prolixicin* transcript expression increased (2.32-fold, SE 1.87–2.63, *P* < 0.0036) (Fig. 3).

### The mortality of Tp***toll***^***−***^***/***Tp***pgrp-lc***^−^-***silenced*** insects increased after challenges with ***E. coli*** and ***M. luteus***

**Fig. 4:**
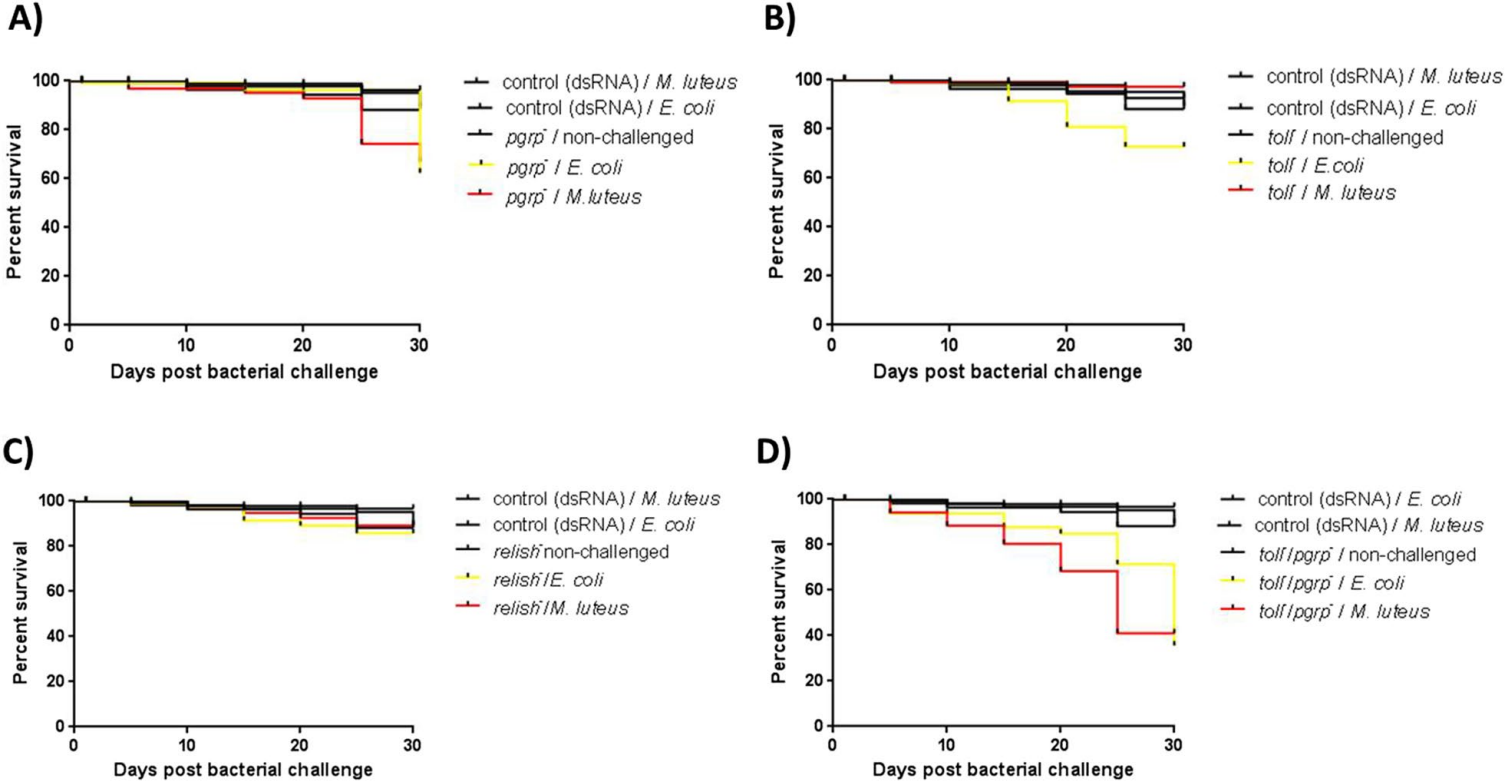
Survival of Tp*pgrp-lc*-, Tp*toll*-, Tp*relish*-, and Tp*pgrp-lc*/Tp*toll*-silenced *T. pallidipennis* after challenge with *E. coli* and *M. luteus*. **a** Bugs inhibited in Tp*pgrp-lc* and challenged with *E. coli* or *M. luteus*. **b** Bugs inhibited in Tp*toll* and challenged with *E. coli*. **c** Bugs inhibited in Tp*relish* and challenged with *E. coli*. **d** Bugs inhibited in Tp*pgrp-lc*/Tp*toll* and challenged with *E. coli* or *M. luteus*. Bugs that received *irrelevant dsRNA* and were challenged with *E. coli* or *M. luteus* or were not challenged served as a control. Statistical analysis of survival analysis was carried out based on Kaplan–Meier plots (log-rank Chi-square test; *P < 0.0001)

The survival after 30 days of observation of non-challenged and Tp*pgrp-lc*-, Tp*relish*-, or Tp*toll*-silenced insects was similar to that of the controls inoculated with *irrelevant dsRNA* (96, 92, and 96%, *P* < 0.05, respectively). After the *E. coli* challenge, survival diminished in Tp*pgrp-lc*, p*toll*, and Tp*relish*-silenced groups (62.3, 72.8, and 85.7%, *P* < 0.05, respectively) (Fig. 4a–c). After the *M. luteus* challenge, survival diminished in the Tp*pgrp-lc*-silenced group (66.8%, *P* < 0.05) (Fig. 4a). Decreased survival was more notable 30 days post-inoculation in groups of bugs with both receptors (Tp*pgrp-lc* and Tp*toll*) silenced and challenged with *E. coli* (35.7%) or *M. luteus* (41%) (*P* < 0.0001) (Fig. 4d).

## Discussion

We documented the participation of the Tp*pgrp-lc* and Tp*toll* receptors, as well as the Tp*relish* transcription factor, in the activation of the IMD and Toll pathways in the immune response of *T. pallidipennis* to *M. luteus* (Gram-positive) and *E. coli* (Gram-negative) bacteria. The intensity of the induction of Tp*pgrp-lc*, Tp*toll*, and Tp*relish* and the AMP *defensin B*, *prolixicin*, and *lysozyme B* transcripts was higher in blood-fed insects, indicating possible participation of the digestive tract microbiota in the immune response, which increased after blood-feeding [39]. Our results indicate that the activation of Tp*toll* (Toll pathway) was of greater intensity against *M. luteus*, while the activation of Tp*pgrp-lc* and Tp*relish* (IMD pathway) was of greater intensity against *E. coli*. This resulted in higher activation of the corresponding specific AMP transcripts, as in *D. melanogaster* [30].

Our results confirm that in *T. pallidipennis*, the orthologous receptor Tp*pgrp-lc* participates in the activation of the IMD pathway and the induction of AMP transcripts. However, this receptor seems to interact with the Toll pathway, as its interference significantly reduced the expression of *defensin B*, *prolixicin*, and *lysozyme B* transcripts after challenge with *M. luteus*.

It was previously reported that in *R. prolixus*, the inhibition of *pgrp-lc-la* decreased the expression of *defensin B*, *lysozyme B*, and *prolixicin* transcripts (IMD-regulated) after a challenge with *E. coli*, but no effect was observed after a challenge with Gram-positive bacteria (*Staphylococcus carnosus*) [36], suggesting that there was specificity in the immune pathway activation. However, we found that in *T. pallidipennis* challenged with *E. coli*, silencing of Tp*pgrp-lc* resulted in decreased expression of AMP transcripts regulated by the IMD pathway, but it also produced a reduction in expression of *defensin B*, *lysozyme B*, and *prolixicin* transcripts after a challenge with *M. luteus,* suggesting that isoforms of Tp*pgrp-lc* could participate specifically in response to Gram-positive bacteria.

On the other hand, silencing of Tp*toll* in *T. pallidipennis* decreased the expression of *prolixicin* and *lysozyme B* after challenge with *E. coli*, and of *defensin B* and *lysozyme B* transcripts in insects challenged with *M. luteus*. These observations suggest that Toll receptors participate in the generation of immune responses against both Gram-positive and Gram-negative bacteria, which indicates that, as in *D. melanogaster* [40, 41], other toll receptors could mediate the activation of immune responses against Gram-negative bacteria [41]. This possible participation of other Toll receptors in triatomines warrants further investigation.

Silencing *relish* in *R. prolixus* challenged with Gram-negative *Enterobacter cloacae* or Gram-positive *Staphylococcus aureus* resulted in reduced levels of *prolixicin* and *defensin A*, *B*, and *C* transcripts [42]. Accordingly, *defensin* and *prolixicin* transcript expression decreased in response to the *relish* inhibitor IMD-0354 [43]. In our handswith our experiments and in our model, Relish appears to participate in both IMD and toll pathways, Relish appears to be involved in both IMD and Toll immune cascades, as silencing of Tp*relish* decreased the transcription of *lysozyme B* in *E. coli*-challenged and *defensin B* and *prolixicin* in *M. luteus*-challenged *T. pallidipennis*, and *relish* was also induced by *M. luteus*, adding support to an interaction between the IMD and Toll pathways and suggesting cross-talk between the immune pathways, as has been suggested in other insect models [27, 30].

**Fig. 5:**
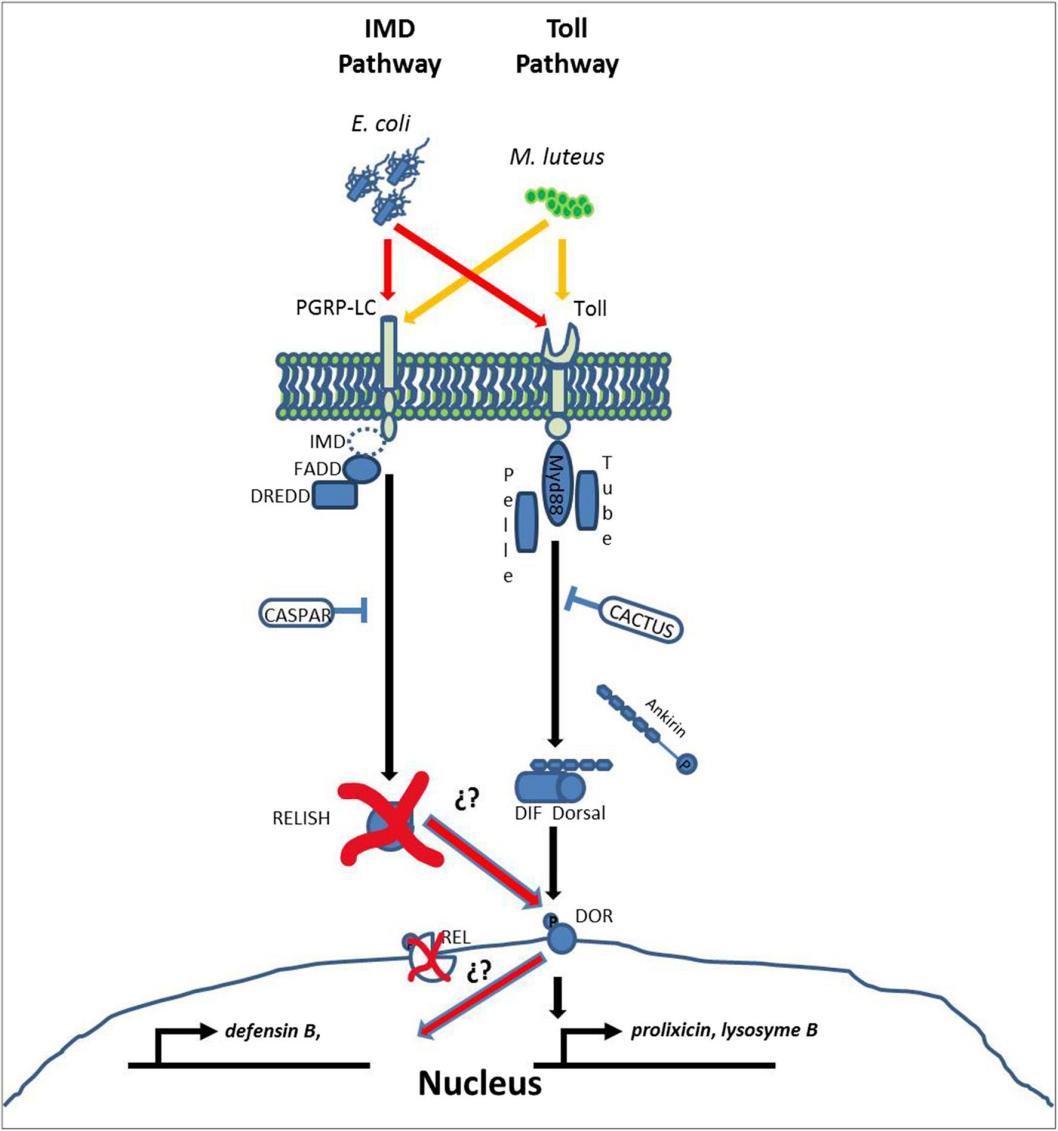
Model proposing the participation of Dorsal transcription factor to compensate for the absence of Tp*relish*. Although the expression of Tp*relish* decreased upon inoculation of dsTp*relish*, *defensin B* transcript increased, which may be due to the participation of other transcription factors such as Dorsal or other molecules or mechanisms that are activated after challenge with *E. coli* or *M. luteus*

In contrast, an increase in *prolixicin* and *defensin B* transcription occurred after we silenced Tp*relish* in *T. pallidipennis* challenged with *E. coli*, which is consistent with observations in *Rhynchophorus ferrugineus* (Coleoptera: Dryophthoridae) challenged with *E. coli*, where *relish* knockdown increased the expression of *defensin* transcripts [44]. Together, these observations indicate the possibility of the additional participation of other transcription factors, such as dorsal-related immunity factor (DIF)/Dorsal [15]. We could speculate that after activation by *E. coli*, this complex probably receives a signal via the IMD pathway, and then the phosphorylated Dorsal is translocated to the nucleus to activate (without the participation of relish) *defensin B* and *prolixicin* transcripts that are canonically synthesized mainly in response to Gram-negative bacteria (*E. coli*). It has been proposed that DIF may compensate for the lack of *relish* to generate the expression of the AMPs [17], a situation that could occur in *T. pallidipennis* (Fig. 5). However, we cannot rule out the existence of relish isoforms or reactivation of this transcription factor in Tp*relish*-silenced insects after bacterial challenge. We are currently conducting experiments to test this hypothesis.

The decrease in *lysozyme B* and *defensin B* transcription after *relish* inhibition in *T. pallidipennis* challenged with *E. coli* and *M. luteus*, respectively, and the increase in *defensin B* transcripts after *E. coli* challenge [45–47] support the possible participation of *relish* or other *relish* isoforms in the response to Gram-positive bacteria, as observed in Lepidoptera and Diptera insects [45–47]. On the other hand, in *T. pallidipennis*, *relish* was also induced by *M. luteus*, adding support to an interaction between the IMD and Toll pathways, as has been reported in other insects [27, 30]. We only analyzed the expression of some immune peptide transcripts, however, and there are most likely more isoforms of these peptide transcripts and other immune response genes such as attacins and cecropins that should be analyzed for a comprehensive understanding of the immune pathways in triatomines.

Although we did not attempt to recover the inoculated bacteria, the results of the survival trials using Tp*pgrp-lc*-, Tp*toll*-, and Tp*relish*-silenced insects support the participation of IMD and Toll in the immune defense against *E. coli* and *M. luteus*. The lower survival rate observed in bugs silenced in both Tp*pgrp*^−^ and Tp*toll*^−^ indicates that AMPs produced through the activation of either immune pathway could suffice to control bacterial infection; also, the added effect of double silencing provides further support for interactions between the IMD and Toll pathways to produce essential AMPs to eliminate bacteria. These results confirm the participation of Tp*pgrp-lc* and Tp*toll* together in the induction of AMPs to eliminate the inoculated bacterial population. As can be seen in Fig. 4, when silencing each receptor separately, the bugs' survival decreased (Tp*pgrp-lc* = 62.3%, Tp*toll* = 72.8%), but with silencing of both receptors, survival decreased to 35.7% (*E. coli*) or 41% (*M. luteus*). This result allows us to propose that the IMD and Toll pathways could participate jointly to eliminate Gram-positive and Gram-negative bacteria.

In summary, these results indicate that in *T. pallidipennis*, the activation of the IMD and Toll pathways could be preferential towards diverse bacterial species, but there is cross-talk between pathways leading to the production of diverse AMPs. This is the first report on silencing of Toll in triatomines, and contributes to a better understanding of the functionality of both pathways.

## Supplementary Information

**Additional file 1: Table S1.** Oligonucleotide sequences of *T. pallidipennis* transcripts used in this work.

**Additional file 2: Table S2.** Bacterial challenge interfered and survival assays groups.

**Additional file 3: Figure S1.** Inhibition kinetics of Tp*pgrp-lc*, Tp*toll*, and Tp*relish* transcripts in *T. pallidipennis* fifth-instar bugs. Groups of 12 insects were inoculated with 2 µg of dsRNA anti-Tp*pgrp*-*lc*, Tp*toll*, or Tp*relish* The fat body of each group was obtained at 4, 7, 11, and 15 days post-inoculation, total RNA was obtained, and cDNA was generated to analyze the expression of the silenced genes. Tp*pgrp-lc* and Tp*relish* transcription decreased at 15 days, while Tp*toll* transcripts were inhibited at 7 days post-dsRNA inoculation. Relative expression 2^−(∆∆CT)^ describes the quantity of the changes between transcripts. **P < 0.05. ***P < 0.001.

**Additional file 4: Figure S2.** Amino acid alignment of Tp*Pgrp-lc* (**a**), Tp*relish* (**b**), Tp*toll* (**c**), *defensin B* (**d**), *prolixicin* (**e**), and *lysozyme B* (**f**) with orthologs from various insect species. Sequences reported in *T. pallidipennis* by Zumaya-Estrada et al. [35] are partial (Tp*pgrp-lc*: TPAL_isotig03340; Tp*toll*: TPAL_H9TUR5Q01DQBBI; Tp*relish*: TPAL_H9TUR5Q02INIGT; *prolixicin*: TPAL_isotig05995, *defensin B*: TPAL_H9TUR5Q02J2RC5; *lysozyme B*: TPAL_isotig04641). The knocked-down sequences of each transcript are marked in a solid red line; the sequences analyzed by qPCR are shown in dotted red lines. *R. prolixus* (*Rhodnius prolixus*), *D. melanogaster* (*Drosophila melanogaster*), *P. stali* (*Plautia stali*), *C. lectularius* (*Cimex lectularius*), *T. brasiliensis* (*Triatoma brasiliensis*), *R. speratus* (*Reticulitermes speratus*), *C. formosanus* (*Coptotermes formosanus*). Black box: **a** *pgrp-lc*: *N*-acetylmuramoyl-l-alanine amidase-like domain. **b** *Relish*: nuclear factor NF-kappa-B protein, **c** *toll*: toll/interleukin receptor TIR domain, **d** *defensin B*: defensin invertebrate/fungal domain, **e** *prolixicin*: attacin C domain, **f** *lysozyme B*: lysozyme-like domain. Blue arrow in **a**: transmembrane domain.

## Data Availability

The datasets supporting the conclusions of this article are included within the article and its additional files.

## Abbreviations

*pgrp-lc*: Peptidoglycan recognition protein-long chain
AMPs: Antimicrobial peptides
IMD: Immune deficiency
DIF: Dorsal-related immunity factor
dsRNA: Double-stranded RNA
DREDD: Death-related ced-3/Nedd2-like caspase
MyD88: Myeloid differentiation primary response protein
NF-κB: Nuclear factor kappa B
FADD: Fas-associated death domain protein
TAK1: Transforming growth factor beta-activated kinase-1
IKK: IκB kinase
