# Supplementary figures and images for: The IMD and Toll canonical immune pathways of Triatoma pallidipennis are preferentially activated by Gram-negative and Gram-positive bacteria, respectively, but cross-activation also occurs
Source: Parasit Vectors. 2022 Jul 12;15:256. doi: 10.1186/s13071-022-05363-y (PMC9277830; doi:10.1186/s13071-022-05363-y)

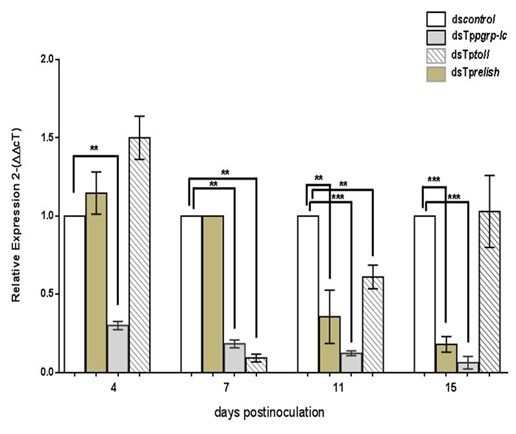

Supplement: Supplementary file 3 — Additional file 3: Figure S1. Inhibition kinetics of Tppgrp-lc, Tptoll, and Tprelish transcripts in T. pallidipennis fifth-instar bugs. Groups of 12 insects were inoculated with 2 µg of dsRNA anti-Tppgrp-lc, Tptoll, or Tprelish The fat body of each group was obtained at 4, 7, 11, and 15 days post-inoculation, total RNA was obtained, and cDNA was generated to analyze the expression of the silenced genes. Tppgrp-lc and Tprelish transcription decreased at 15 days, while Tptoll transcripts were inhibited at 7 days post-dsRNA inoculation. Relative expression 2−(∆∆CT) describes the quantity of the changes between transcripts. **P < 0.05. ***P < 0.001. [file 13071_2022_5363_MOESM3_ESM.jpg]

A)

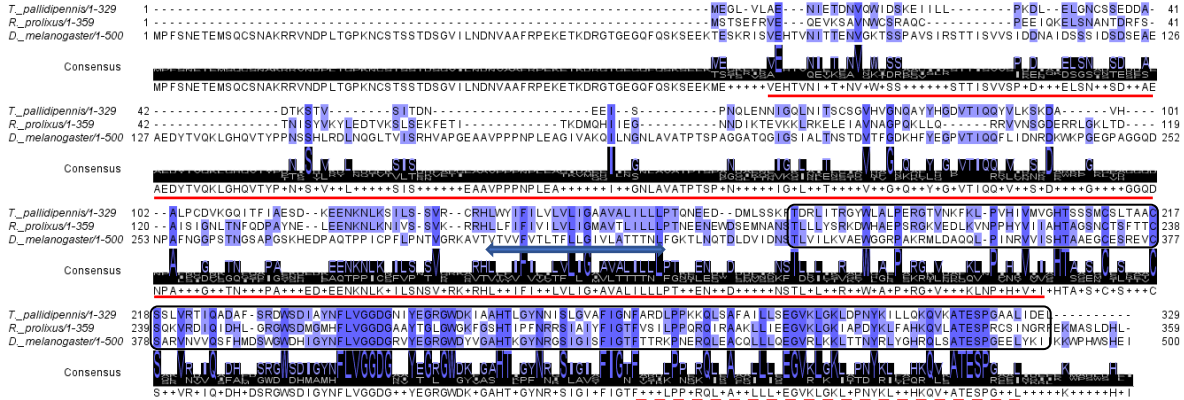

B)

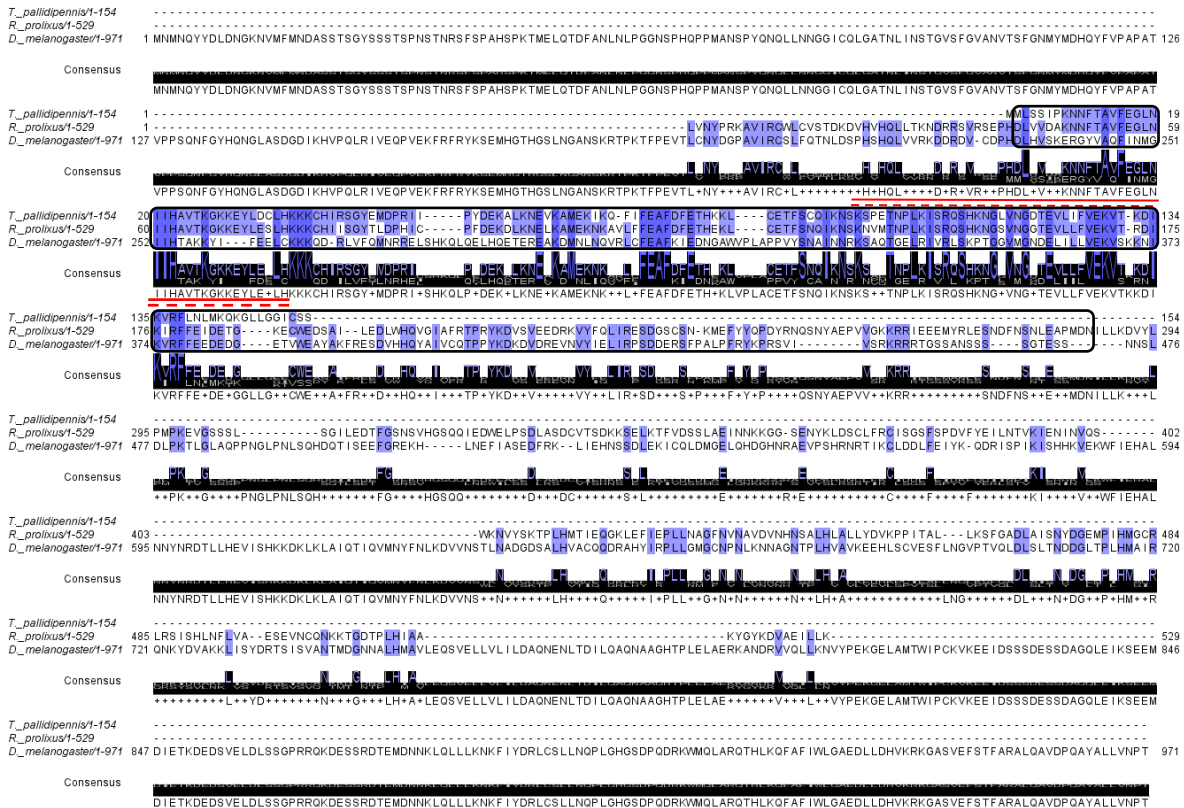

C)

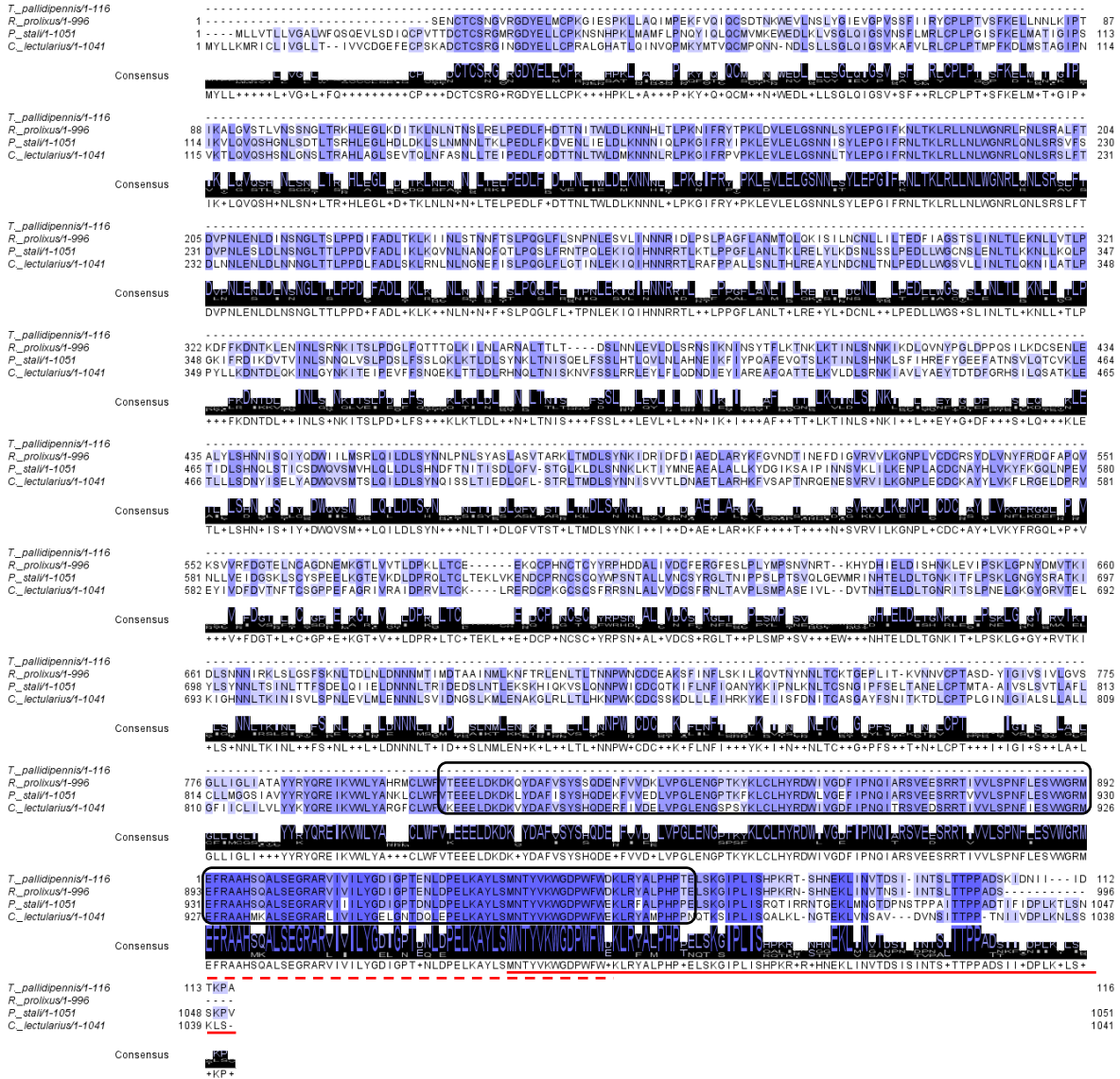

D)

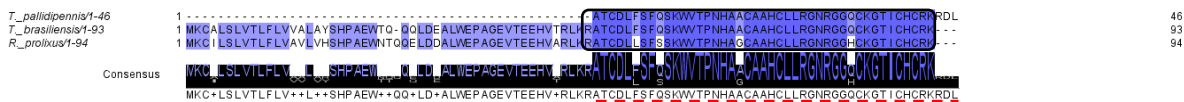

E)

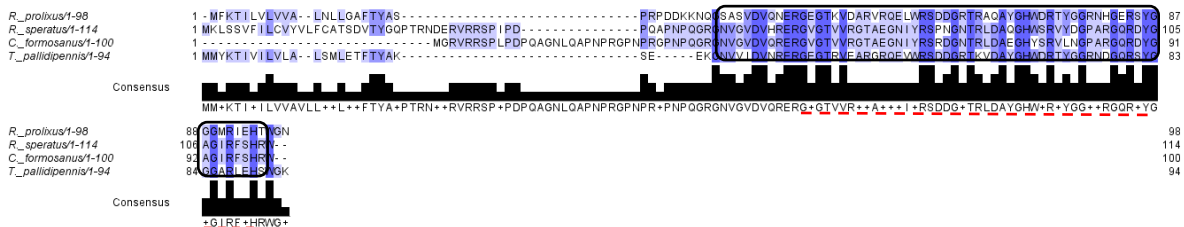

F)

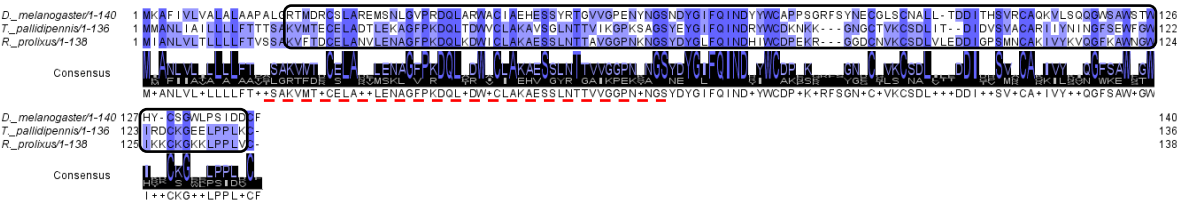

Supplement: Supplementary file 4 — Additional file 4: Figure S2. Amino acid alignment of TpPgrp-lc (a), Tprelish (b), Tptoll (c), defensin B (d), prolixicin (e), and lysozyme B (f) with orthologs from various insect species. Sequences reported in T. pallidipennis by Zumaya-Estrada et al. [35] are partial (Tppgrp-lc: TPAL_isotig03340; Tptoll: TPAL_H9TUR5Q01DQBBI; Tprelish: TPAL_H9TUR5Q02INIGT; prolixicin: TPAL_isotig05995, defensin B: TPAL_H9TUR5Q02J2RC5; lysozyme B: TPAL_isotig04641). The knocked-down sequences of each transcript are marked in a solid red line; the sequences analyzed by qPCR are shown in dotted red lines. R. prolixus (Rhodnius prolixus), D. melanogaster (Drosophila melanogaster), P. stali (Plautia stali), C. lectularius (Cimex lectularius), T. brasiliensis (Triatoma brasiliensis), R. speratus (Reticulitermes speratus), C. formosanus (Coptotermes formosanus). Black box: a pgrp-lc: N-acetylmuramoyl-l-alanine amidase-like domain. b Relish: nuclear factor NF-kappa-B protein, c toll: toll/interleukin receptor TIR domain, d defensin B: defensin invertebrate/fungal domain, e prolixicin: attacin C domain, f lysozyme B: lysozyme-like domain. Blue arrow in a: transmembrane domain. [file 13071_2022_5363_MOESM4_ESM.pdf]
